# **Assessment of circularized E7 RNA, GLUT1, and PD-L1 in anal squamous cell carcinoma**

**DOI:** 10.18632/oncotarget.27234

**Published:** 2019-10-15

**Authors:** Bahir H. Chamseddin, Eunice E. Lee, Jiwoong Kim, Xiaowei Zhan, Rong Yang, Kathleen M. Murphy, Cheryl Lewis, Gregory A. Hosler, Suntrea T. Hammer, Richard C. Wang

**Affiliations:** ^1^Department of Dermatology, UT Southwestern Medical Center, Dallas, 75390, TX, USA; ^2^Department of Clinical Science, UT Southwestern Medical Center, Dallas, 75390, TX, USA; ^3^ProPath Dermatopathology, Dallas, 75247, TX, USA; ^4^Harold C. Simmons Center, UT Southwestern Medical Center, Dallas, 75390, TX, USA; ^5^Department of Pathology, UT Southwestern Medical Center, Dallas, 75390, TX, USA; ^*^These authors contributed equally to this work

**Keywords:** anal squamous cell carcinoma, circular RNA, GLUT1, human papillomavirus, PD-L1

## Abstract

**Anal squamous cell carcinoma (ASCC) is a rare, potentially fatal malignancy primarily caused by high-risk human papillomaviruses (HPV). The prognostic implication of programmed death-ligand 1 (PD-L1) expression remains controversial, and glucose transporter 1 (GLUT1) expression has never been examined in ASCC. Covalently closed circular RNAs have recently been shown to be widespread in cancers and are proposed to be biomarkers. We discovered HPV16 expresses a circular E7 RNA (circE7) which has not been assessed as a potential biomarker. A retrospective, translational case series at UT Southwestern was conducted to analyze PD-L1, GLUT1, HPV-ISH, and HPV circE7 in relation to the clinical features and overall survival of patients with ASCC. Twenty-two (22) subjects were included in the study. Improved overall survival was predicted by basaloid histology (** *p* **= 0.013), PD-L1 expression (** *p* **= 0.08), and HPV-ISH positivity (** *p* **& 0.001), but not GLUT1 expression. High levels of circE7 by quantitative RT-PCR predicted improved overall survival in ASCC (** *p* **= 0.023) and analysis of The Cancer Genome Atlas sequencing from HPV-positive head and neck cancer and cervical cancer suggested high circE7 marked improved survival in 875 subjects (** *p* **= 0.074). While our study suggests that circE7 levels correlate with improved survival in ASCC, larger, prospective studies are necessary to confirm the potential role of circE7 as a biomarker.** ****

## **Introduction**

Anal Squamous Cell Carcinoma (ASCC) has 30,000 new cases diagnosed annually worldwide and the incidence is rising by 2% each year [1]. A major risk factor is immunosuppression, which may be iatrogenic (transplant patients) or secondary to infection with the Human Immunodeficiency Virus (HIV) [2]. Other risk factors for ASCC include male-to-male sex, a high-life time number of sexual partners, and cigarette smoking [3]. Infection by high-risk human papillomavirus (HPV) is causative, and, in many cases, is also linked to immunosuppression. The most effective treatment for advanced locoregional or metastatic ASCC is Mitomycin C and 5-Fluorouracil or Cisplatin with concurrent radiotherapy, known as the Nigro Protocol. Surgical excision may be employed yet 30% of cases do not respond or relapse locally [4]. Over 40 years, the standard Nigro Protocol has shown a 5-year survival of ~60% in metastatic cases of ASCC [1, 5].

Investigations for both novel therapies and biomarkers in ASCC are necessary. Past studies have shown that epidermal growth factor receptor (EGFR) overexpression occurs in almost all cases of ASCC [6]. Clinical trials with Cetuximab, a EGFR-monoclonal antibody, proved to be toxic and did not progress [7, 8]. Programmed death ligand-1 (PD-L1), a marker of immunogenicity, has been investigated in patients with ASCC and positive expression correlated with an improved prognosis [9]. However, this finding is in conflict with several other recent studies [10, 11]. One prospective clinical trial of a “checkpoint inhibitor” targeting the PD-1 receptor (e.g. pembrolizumab) in treatment-refractory ASCC did not yield curative outcomes in a majority of patients [12]. However, there are ongoing clinical trials for checkpoint (anti-PD-1/PD-L1) inhibitors including Nivolumab and Pembrolizumab for different stages of ASCC (NCT02919969, NCT03233711).

The facilitative glucose transporter 1 (GLUT1) regulates glucose transport in the skin, especially keratinocytes. The transporter may play an important role in sustaining carcinogenesis by fueling anaerobic glycolysis in the hypoxic environment of the tumor [13]. HPV-driven cancers such as head and neck squamous cell cancers show an overexpression of GLUT1 but the relationship between HPV and the GLUT1 transporter has never been explored in ASCC [14, 15]. Due to the development of ASCC from keratinocytes and the high incidence of HPV, the expression of this transporter should be explored in the context of ASCC and HPV infection.

High-risk human papillomaviruses (HPVs), especially HPV16, is implicated in 70-90% of cases of ASCC and is a primary driver of oncogenesis [16]. High-risk HPVs encode for the E6 and E7 oncoproteins, which disrupt the p53 and Rb tumor suppressor checkpoints, respectively, and promote cell proliferation and ultimately the development of cancer [17]. Recent studies have unexpectedly revealed that HPV positive cancers possess abundant single-stranded, circular RNAs (circRNAs) derived from viral sequences [18]. Specifically, a circular RNA from HPV16 that contains the entire E7 oncoprotein open reading frame (circE7) has recently been described. The stability and regulation of circE7 appears to be unique compared to linear HPV RNAs like E6*I, which can also encode for the E7 oncoprotein. CircRNAs have been reported to be more stable than linear mRNAs and preferentially packaged in exosomes and, thus, could contribute to the pathogenesis of HPV-mediated infection and tumorigenesis in unique ways [19]. As circular RNAs are abundant and appear to have distinct functional properties compared to their linear counterparts, it will be important to determine whether cancer-derived circRNAs have diagnostic potential for some cancers [20, 21].

In this report, we examine both established (i.e., PD-L1 and GLUT1) and novel (i.e. HPV16 circE7) biomarkers and their role in prognosis in HPV-driven ASCC. We also correlate these findings to demographic, clinical, and prognostic factors in these patients. We identify high levels of circE7 as a positive prognostic marker in ASCC, and potentially other HPV-driven cancers.

## **Results**

### Clinical data and overall survival

Of the 22 patients with ASCC included in the study for clinical analysis, 18 cases had tissue available for molecular studies and 16 had available tissue for immunohistochemistry and *in-situ* hybridization. A schematic of the study design is included in the Supplementary Figures. The demographic, clinical, and histopathologic characteristics of patients who underwent molecular studies are listed in Table 1. The average age of the subjects was 61.5 (±7.7), 13 (59%) of the subjects were female, 19 (86%) were Caucasian, and 3 (14%) were African American. Patients with tissue that underwent immunohistochemical analysis had an average age of 61.7 (±8.18), female count of 10 (63%), Caucasian ethnicity count of 15 (95%), and African American ethnicity count of 1 (5%). Clinically, lower overall survival rate could be predicted by male sex (hazard ratio (HR): 4.04, 95% confidence interval (CI): 1.2-13.5, *p* = 0.034), higher T stage (HR: 4.09, CI: 1.3-12.7, *p* = 0.046), and higher N stage (HR: 9.31, CI: 2.8-30.4, *p* = 0.008). Decreased overall survival could also be predicted by greater smoking pack-years (HR: 5.88, CI: 1.8-19.3, *p* = 0.01) and greater size of tumor (>5 cm dimension) upon presentation (HR: 3.59, CI:1.1-11.5, *p* = 0.03) (Table 2). Patients who underwent surgery had a poorer overall survival than those treated with chemotherapy alone (HR:3.89, CI: 1.1-13.9, *p* = .0375), independent of other factors listed above. Age, ethnicity, medical comorbidities, chemotherapeutic agent, and use of radiotherapy did not correlate to prognosis.

**Table 1:** Demographic, clinical, and histologic features of patients with anal squamous cell carcinoma

Sample	Age	Sex	Ethnicity	Smoking	T	N	M	Size (cm)	Treatment	Architecture	Grade	GLUT1	PD-L1	HPV-ISH	circE7	OS
*1*	70.3	F	C	0	2	0	0	2.6	CR	Basaloid	3	High	+	+	Low	3.75
*2*	64.1	F	C	50	4	3	1	8	O	Basaloid	3	Low	+	+	Low	^*^0.41
*3*	74.3	F	C	30	3	0	1	3.1	CRS	Mixed	2	High	+	+	High	4.49
*4*	60.6	M	C	40	4	0	1	8	CS	Mixed	2	Low	-	+	Low	^*^1.68
*5*	63.0	F	C	15	2	0	0	3	CR	Basaloid	3	Low	+	+	High	5.24
*6*	69.9	M	C	25	3	1	1	8	CS	Basaloid	2	Low	+	+	High	^*^2.38
*7*	66.9	M	C	30	4	0	1	7.3	CRS	Keratinizing	1	High	+	+	High	^*^5.69
*8*	60.9	M	C	0	3	0	0	6.7	CRS	Mixed	2	Low	-	+	Low	^*^2.21
*9*	50.2	F	C	0	1	2	0	1.5	CR	Basaloid	3	High	+	+	High	5.78
*10*	68.3	F	C	2.5	2	0	0	5	CR	Basaloid	3	Low	-	+	Low	2.48
*11*	57.5	F	C	0	1	1	0	1.6	CR	Basaloid	3	Low	+	+	High	4.57
*12*	49.8	F	C	10	1	0	0	1.5	S	Basaloid	2	High	+	+	High	6.27
*13*	49.6	F	C	36	3	1	0	10.5	CRS	Keratinizing	2	Low	-	-	Low	^*^2.83
*14*	51.2	M	C	20	4	2	0	4.6	CRS	Keratinizing	1	High	-	-	Low	^*^1.7
*15*	60.2	M	C	60	3	3	0	12	CRS	Mixed	2	High	-	+	Low	^*^0.88
*16*	70.5	F	AA	0	4	1	0	3.2	CRS	Mixed	2	High	+	+	High	^*^1.36
*17*	53.0	M	AA	19	2	0	0	4.4	CRS	n.d.	n.d.	n.d.	n.d.	n.d.	High	0.5
*18*	53.0	F	AA	0	2	1	1	6.8	CRS	n.d.	n.d.	n.d.	n.d.	n.d.	Low	^*^2.75

Abbreviations: F, female; M, male; C, Caucasian; AA, African American; T, tumor stage; N, node stage; M, metastasis stage; CRS, neoadjuvant chemoradiotherapy and surgery; CS, neoadjuvant chemotherapy and surgery; CR, neoadjuvant chemoradiotherapy; S, surgery alone; O, no treatment; OS = Overall Survival Time (years); n.d. – not determined; ^*^ - Death occurred at time since initial treatment.

**Table 2:** Clinical and biomarker characteristics for overall survival in patients with anal squamous cell carcinoma

Overall survival	Univariate		
	*p*-value	HR	95% CI
Age (>/59 y)	0.212	0.40	0.12 to 1.33
**Sex (male / female)**	**0.034**	4.05	1.22 to 13.47
**T-category (T3–4 / T1–2)**	**0.046**	4.09	1.31 to 12.75
**N-category (N1–3 / N0)**	**0.008**	9.32	2.86 to 30.40
M (1 / 0)	0.051	3.50	0.99 to 12.27
**Smoking (median, 17.4 py)**	**0.009**	5.88	1.79 to 19.39
**Size (median, 5.3 cm)**	**0.031**	3.59	1.12 to 11.50
Grade (3&4 / 1&2)	0.101	3.27	0.79 to 13.51
**Keratinizing & Mixed / Basaloid**	**0.014**	8.89	1.96 to 40.23
GLUT-1(>/median)	0.421	1.26	0.31 to 5.12
PD-L1 (+ / –)	0.084	0.17	0.03 to 0.82
HPV16-ISH (+ / –)	0.318	0.35	0.04 to 2.74
**circE7 (high / low)**	**0.026**	0.14	0.05 to 0.72
Linear E6^*^1 (high / low)	0.290	2.05	0.04 to 0.47

HR, hazard ratio; CI, confidence interval; T, tumor stage; N, node stage; M, metastasis stage; py, pack years.

### Histologic features and overall survival

**Figure 1:**
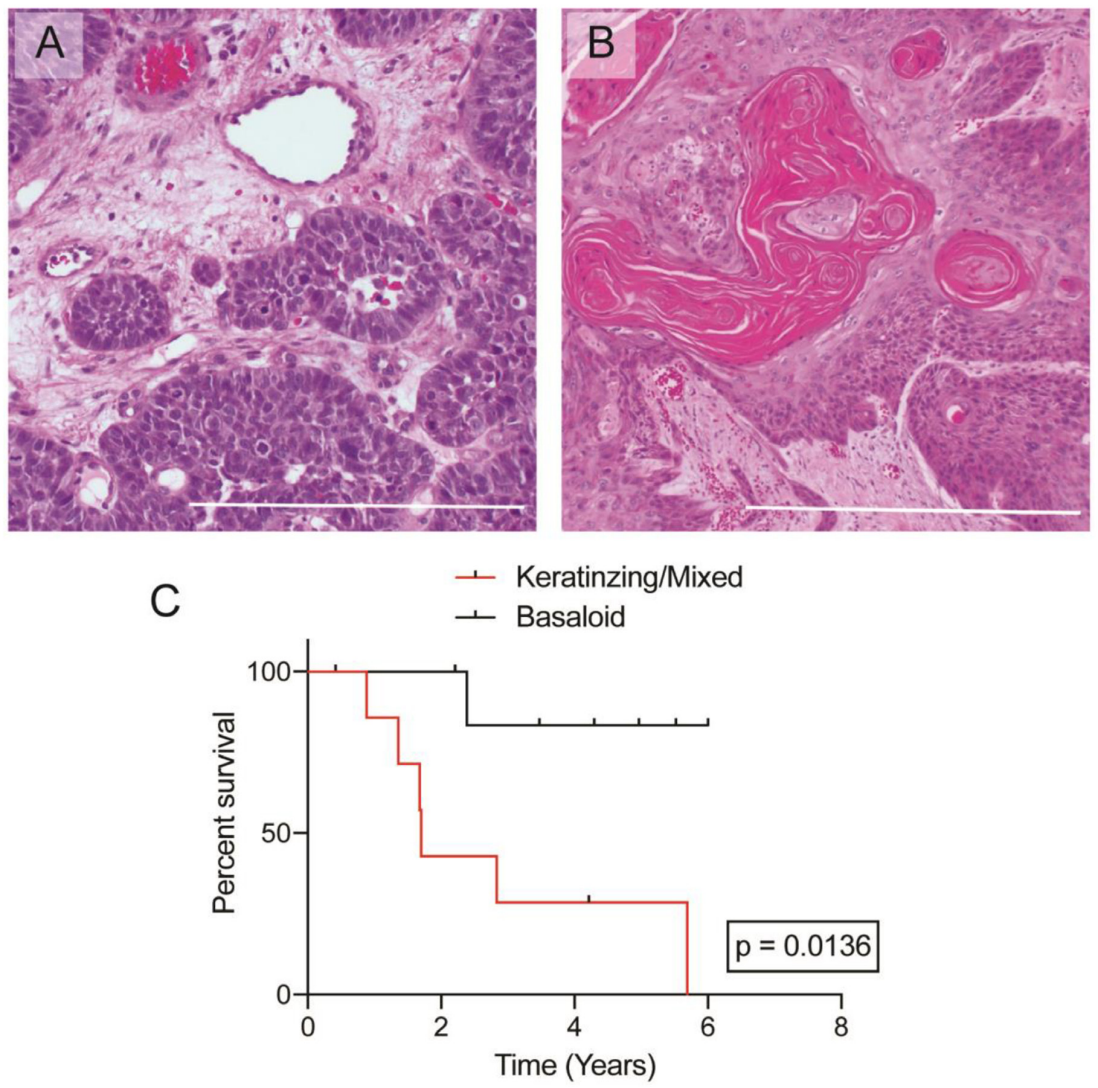
Histologic images of anal squamous cell carcinoma. (**A**) Representative image of a “basaloid” architecture in ASCC, 100X. (**B**) Representative image of a “keratinizing” architecture in ASCC, 100X. (**C**) Kaplan–Meier curves plotting overall survival based on histologic architecture. Bar = 500 **μ**m.

A basaloid architecture was present in 9 (56%) of the biopsies as the sole architecture (Figure 1A). Three (16%) tumors had a purely keratinizing architecture (Figure 1B). Notably, two of the three keratinizing tumors were HPV-negative. Four (25%) biopsies showed a mixed phenotype. The presence of any keratinizing features, including both keratinizing and mixed histologic phenotypes, correlated to a lower OS compared to the purely basaloid subtype in this cohort (*p* = 0.013) (Figure 1C). Architecture type was independent of any clinical features related to prognosis. Higher tumor grade, presence of perineural invasion, lymphovascular invasion, necrosis, and inflammation were not significantly correlated to clinical outcomes.

### Biomarker analysis, clinical characteristics, and overall survival

**Figure 2:**
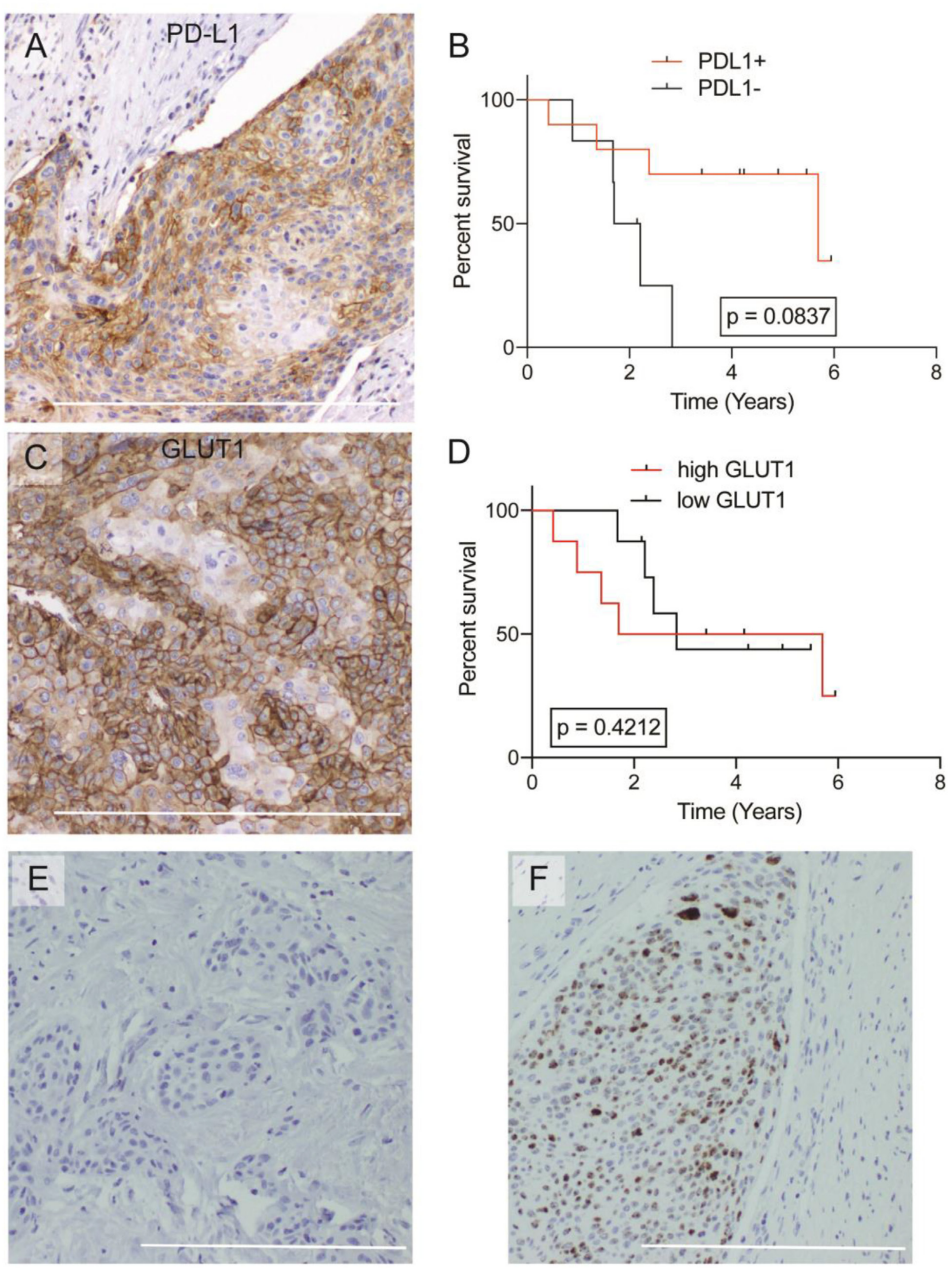
Overall survival analysis of GLUT1, PD-L1, and histologic architecture. (**A**) Representative image of IHC for PD-L1+ ASCC, 200X. (**B**) Kaplan–Meier curves plotting overall survival based on PD-L1 staining. (**C**) Representative image of IHC for GLUT1+ ASCC, 200X. (**D**) Kaplan–Meier curves plotting overall survival based on GLUT1 staining. (**E**) Representative image of HPV-ISH negative tumor, 100X. (**F**) Representative image of HPV-ISH positive tumor, 100X. Bar = 500 **μ**m.

Biomarker and histologic features were analyzed for relationships (Table 3). There were no differences between any of the potential biomarker groups with respect to age, gender, ethnicity, smoking pack years, tumor stage, or treatments. Ten (10) of the 16 samples were positive for PD-L1 (Figure 2A), which positively correlated with HPV-ISH positivity (*p* & 0.001), high circE7 levels (*p* = 0.038), and smaller tumor size (*p* = 0.020) (Table 3). PD-L1 positivity trended towards improved overall survival, though it did not reach significance (*p* = 0.0837) (Figure 2B). All 16 samples were positive for GLUT1 expression which stained the tumor cell membranes (Figure 2C). GLUT1 was quantified by a weighted score and divided into high and low expression by the median values. GLUT1 expression levels did not correlate to any significant findings in clinicopathologic characteristics including overall survival (Table 3, Figure 2D). HPV-ISH signal was not detected in 2 of the 16 samples (Figure 2E–2F); these negative samples correlated to the pure keratinizing subtype. HPV-ISH negativity correlated to a significantly poorer prognosis (*p* & 0.001) (Table 3).

**Table 3:** Histologic and biomarker (CircE7, GLUT-1, and PD-L1) features in patients with anal squamous cell carcinoma

	**Total** **(*n* = 16)**	**High CircE7 (*n* = 8)**	**Low CircE7 (*n* = 8)**	***p*-value **	**High GLUT1 (*n* = 8)**	**Low GLUT1 (*n* = 8)**	***p*-value **	**PD-L1 + (*n* = 10)**	**PD-L1 - (*n* = 6)**	***p*-value **
Grade (I/II)	10	4	6		6	4		5	5	
Grade (III/IV)	6	4	2	*0.3*	2	4	*0.3*	5	1	*0.18*
Keratinizing/Mixed	7	1	6		5	2		3	4	
Basaloid	9	7	2	***0.01***	3	6	*0.13*	7	2	*0.15*
GLUT1, high	8	3	5		-	-	*-*	6	2	
GLUT1, low	8	5	3	*0.31*	-	-	*-*	4	4	*0.3*
PDL1, +	10	7	3		6	4		-	-	*–*
PDL1, –	6	1	5	***0.04***	2	4	*0.3*	-	-	*–*
HPVISH, +	14	8	6		7	7		10	4	
HPVISH, –	2	0	2	*** &0.001***	1	1	*1*	0	2	*** &0.001***
CircE7, high	8	–	–	*-*	3	5		7	1	
CircE7, low	8	–	–	*-*	5	3	*0.45*	3	5	***0.04***

Mean (SD), Count (Percentage), Bold is significant.

GLUT1, glucose transporter-1, and CircE7 high and low designated based on median value.

PD-L1, programmed death-ligand 1, positive (+) is >1%.

HPV-ISH, human-papilloma virus *in-situ* hybridization, positive (+) is >1%.

Table includes patients and tissues with immunohistochemical staining only.

**Figure 3:**
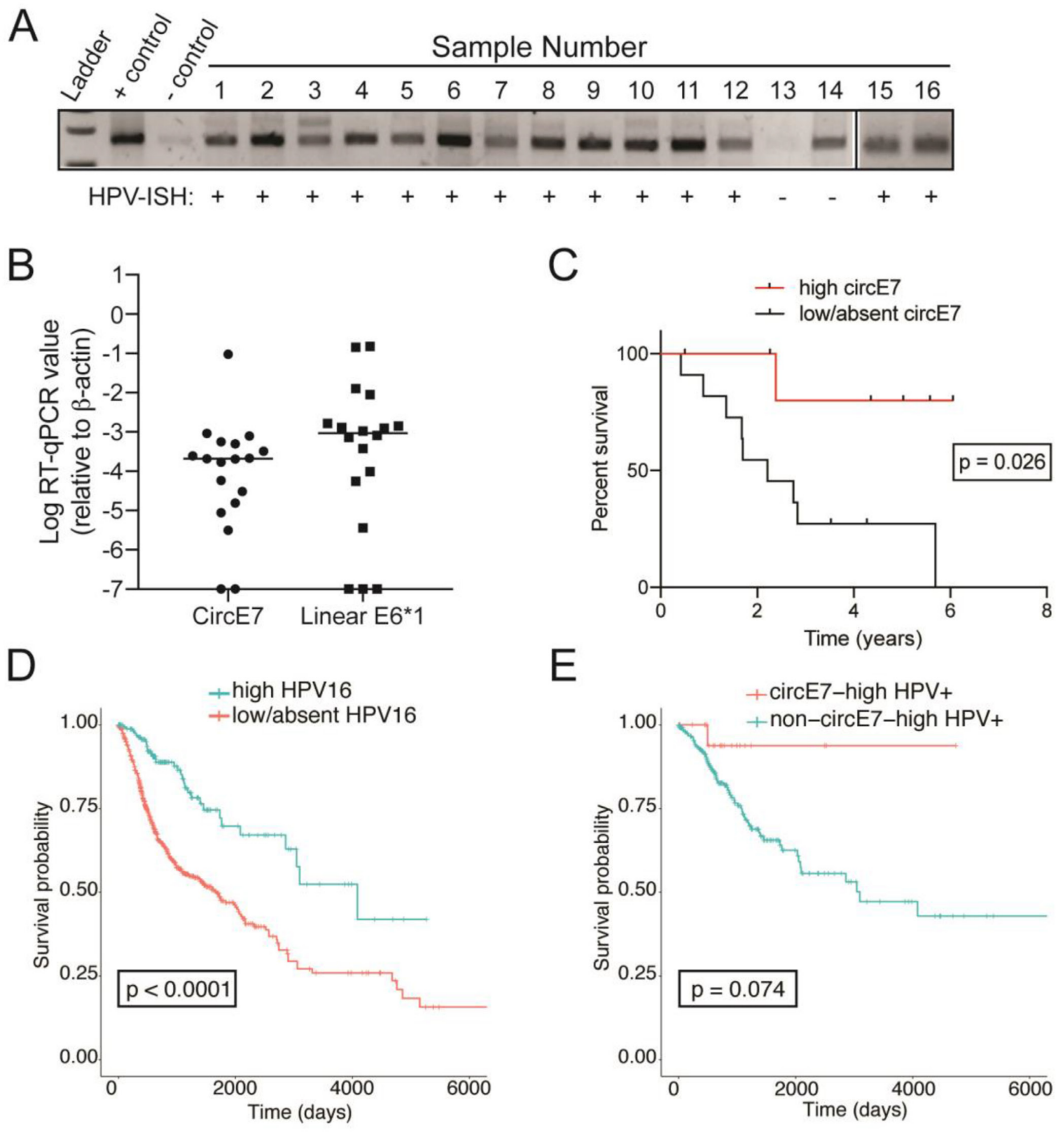
Human papillomavirus circular-E7 RNA analysis in patients with ASCC. (**A**) Endpoint RT-PCR from RNA prepared from FFPE ASCC cases. The presence of multiple bands indicate the presence of distinct circE7 isoforms generated by multiple backsplice events. HPV-ISH status indicated below the image. Controls represent cDNA prepared from 293T cells transiently transfected with a circE7-generating plasmid (+control) of a pcDNA vector (-control). (**B**) Levels of circE7 RNA and Linear E6*I RNA, the linear mRNA transcript for E7, normalized to β-actin levels. Line indicates the median value. (**C**) Kaplan–Meier curves plotting overall survival based on circE7 levels. (**D**) Kaplan–Meier analyses of TGCA RNA-Seq data from head and neck cancer (HSNC) and cervical cancer (CESC) shows that high HPV16 levels (>0.0001 read ratio to HPV16, blue) portends better prognosis in the TCGA patients. (**E**) Kaplan–Meier analyses of TGCA RNA-Seq showing a trend towards improved survival by high circE7 (red) with non-circE7-high tumors (blue).

Endpoint RT-PCR confirmed that inverse primers specific to circE7 amplified products of the expected size (Figure 3A). Some samples displayed multiple circE7 discrete bands, with the higher molecular weight bands consistent with the presence of larger isoforms of circE7 [18]. Next, levels of circE7 and linear E6*I were assessed by RT-qPCR and normalized to levels of β-actin (Figure 3B, Supplementary Figure 1A). The level of circE7 in nine subjects (50%) were categorized as ‘high circE7’ by being higher than the median value of all circE7 values. The remaining samples (9) (50%) subjects) were classified as having ‘low/absent circE7’. Linear E6*I was categorized similarly. Of note, improved overall survival rate was significantly associated with higher circE7 values (*p* = 0.023) (Figure 3C). The high circE7 group also correlated to positive HPV-ISH (*p* & 0.001), positive PD-L1 expression (*p* = 0.038), lower T-stage (*p* = 0.038) and towards basaloid architecture. In contrast, linear E6*I levels did not have prognostic significance (*p* = 0.26).

We sought to confirm the potential utility of circE7 as a novel biomarker in an independent dataset. In previous studies, we found that HPVcircE7 could be detected in RNA-Seq data from cervical squamous cell carcinoma and endocervical adenocarcinoma (CESC) and head and neck squamous cell carcinoma (HNSC) from the Cancer Genome Atlas [18]. Consistent with the existing literature, cancers with high levels of HPV16 (>0.0001 read ratio to HPV16) demonstrated improved survival in oropharyngeal and cervical cancer (*p* = &0.0001) (Figure 3D). Of note, cancers with high levels of circE7 (>30 read count and back-splice junction ratio >0.2) also showed a trend towards improved survival when compared HPV16+ tumors with low or absent circE7 (Figure 3E). In contrast, in the TCGA data, levels of circE7 expression were not correlated with PD-L1, GLUT1, and HPV16 E6*I expression levels. Moreover, none of those factors showed a similar impact on survival (Supplementary Figure 2). Thus, in distinct HPV16+ tumor types, high levels of circE7 as assessed by RNA-Seq were associated with improved survival.

## **Discussion**

The aim of this research was to analyze a novel (HPV16 circE7) and established biomarkers (HPV-ISH, PD-L1, and GLUT1) and correlate these markers to clinical outcomes in patients with ASCC. We also demonstrate for the first time that circE7 is stable enough to be detected in formalin fixed paraffin embedded (FFPE) tumor samples. Although this study was not powered to identify the clinical characteristics that may predict poorer prognosis, we found that male sex, smoking, greater size of tumor upon presentation, and higher TNM stage all correlated with poorer prognosis. Although small in size, our retrospective cohort appears to possess clinical prognostic factors consistent with several larger studies in patients with ASCC [2, 22].

Also, consistent with previous reports, we found that the keratinizing subtype was associated with worse prognosis, and the basaloid subtype with better prognosis [23, 24]. Keratinizing squamous cell carcinomas from the perianal region have been reported to have lower rates of association with HPV infection in some studies [23, 24]. Indeed, the presence of circularized HPV circE7 RNA in this study correlated to the undifferentiated, basaloid subtype. Despite the potential utility of this histological feature, the inter-rater reproducibility of subtyping the architecture of ASCC is poor, which has likely limited its adoption as a scoring criterion in the most recent World Health Organization Classifications [25].

PD-L1, an immune-checkpoint inhibitor found on some cancer cells, immune cells, and other normal tissues, suppresses T cell function by interacting with the lymphocyte PD-1 receptor. While expression of immunosuppressive cell surface receptors might be assumed to portend a poorer prognosis in cancer patients, the opposite has been shown in some cancers, including ASCC [26, 27]. Tumor-infiltrating CD8+ T cells upregulate INFγ, which stimulates both target tissue PD-L1 expression and T-cell PD-1 expression [28]. Thus, PD-L1 thus may act as a marker of an existing robust immune response in some cancers. Consistent with previous ASCC studies, we found that PD-L1 expression correlated with improved prognosis and HPV infection. Several studies have analyzed PD-L1 expression in ASCC, but a consensus on the impact of PD-L1 on prognosis is not clear. Govindarajan et al. found PD-L1 expression to portend poorer local control after 5 years in a retrospective study of 41 patients [11]. However, our finding that PD-L1 trended towards improved survival is more consistent with several other studies in ASCC, which found PD-L1 expression to predict improved overall survival and local control [26]. Moreover, these findings are more consistent with the prognostic utility of PD-L1 in other cancer types including oropharyngeal cancer and ovarian cancer [22]. While expression of PD-L1 in ASCC have yielded the expectation that PD-L1 inhibitors might be an effective therapeutic option, early clinical trials using the PD-L1 inhibitor, pembrolizumab, have been disappointing [12].

Tumor cells may exhibit an altered metabolism to support their unregulated proliferation and sustain their greater energy demands for proliferation and metastasis. In fact, the GLUT1 transporter has been shown to be strikingly upregulated in several cancers [29]. The increase in glucose transport is thought to support upregulated anaerobic glycolytic pathways in the hypoxic tumor environment, potentially contributing to a poor prognosis in those patients. While the GLUT1 transporter is abundant in endothelial cells, erythrocytes, and basal keratinocytes, its level of expression is usually low in differentiated epithelial tissues and benign epithelial tumors [30]. This is the first study to analyze GLUT1 as a prognostic indicator in ASCC. While GLUT1 appeared to be present in all cases of ASCC, its levels had no prognostic significance, possibly because relatively high expression was observed in all samples. In two systemic meta-analyses, GLUT1 expression in gallbladder cancer, pancreatic cancer, breast cancer, oral cancer, and lung cancer did correspond to higher tumor grade, lymph node metastasis and larger tumor size, which are all linked to poorer overall survival [29]. However, similar to this ASCC study, GLUT1 expression in various other tumors including laryngeal, colorectal, esophageal, and cervical had no impact on prognosis [29, 31]. It remains unclear why GLUT1 appears to have different prognostic significance in different tumor types.

In general, studies on HPV-associated malignancies reveal a favorable prognosis in HPV-positive carcinomas compared to the HPV-negative counterparts [26, 32, 33]. Multiple mechanisms have been speculated to contribute to this phenomenon including increased antigenic signals from viruses leading to greater immunogenic responses and increased frequency of double-stranded DNA breaks in high burden HPV tumors [34]. In this study, both by HPV-ISH of ASCC and analysis of RNA-Seq of HPV+ cancers, the presence of HPV correlate to improved prognosis. In the ASCC samples, although HPV-ISH, circE7 qPCR, and Linear E6*I qPCR, all confirmed one sample (sample 14) as HPV-negative, there were discrepancies in identifying the other HPV-negative samples (Supplementary Figure 1A). Although technical differences in the sensitivity and specificity of the individual assays could explain the discrepancies, biological differences in the way that circular and linear HPV RNAs are generated could also contribute to their differential detection. Indeed, the finding that there was not a strong correlation between levels of circE7 and linear E6*I supports this possibility (Supplementary Figure 1B). Notably, high levels of circE7 RNA was significantly correlated with improved survival in ASCC. In the same samples, levels of circE7 did not correlate with expression of PD-L1 or GLUT1 (Supplementary Figure 2). Moreover, linear E6*I did not have prognostic significance, suggesting that properties specific to circE7, rather than global HPV RNA expression, were responsible for the clinical correlations. Thus, our studies on circE7 expand our understanding of the role of HPV in tumor formation.

Analysis of RNA-Seq from HPV+ head and neck cancer and cervical cancers from TGCA data showed a similar trend for improved survival in high circE7 tumors. We have found that circE7 can be translated to the E7 oncoprotein and that its circE7 is essential to the transformed behavior of CaSki cervical carcinoma cells *in vitro* and in tumor xenograft studies [18]. While it is perhaps surprising that higher expression of the oncoprotein-encoding circE7 would correlate with a better prognosis, we speculate on several possibilities that could explain this phenomenon. Higher levels of circE7 could allow for higher expression of the E7 oncoprotein; this may, in turn, allow for a more robust host immune response against the HPV cancer in some cases. While promoting tumor initiation, the E7 oncoprotein has been reported to inhibit the invasion and metastasis of advanced tumors by inhibiting the expression of miR-20a [35]. Additional non-coding functions of circRNAs including miRNA sponging, protein sponging, or transcriptional regulation could also have deleterious impacts on tumor growth [19, 36]. Finally, as circE7 production is decreased after calcium-induced differentiation of keratinocytes [18], levels of circE7 could indicate the differentiation state of the tumor (i.e. “basaloid” vs. “keratinizing”) and, thus, be a sensitive “passenger” biomarker of the more benign “basaloid” tumor architecture [21].

CircRNAs have been shown to be more stable than linear mRNAs in multiple studies [19, 21, 37]. Indeed, we were able to detect circE7 robustly in some FFPE samples despite the poor stability of RNA in this setting. Thus, additional studies for HPV circRNAs as a potential biomarker should be considered, especially given the high rate of HPV16 infections in cancers.

Limitations of this study include its small sample size and the retrospective nature of clinical data collection. This cohort also contained a heterogenous sample of ASCC severity ranging from locoregional to metastatic and the data was collected at a large, tertiary care center, and thus may have a selection bias for more severe cases of ASCC. Additionally, the nature of examination of biomarkers from histology may lead to sampling error of the tumor and thus exact measurements of biomarkers present in the sample may differ in regions of the patient’s tumor.

In summary, this retrospective, translational pilot study examined a novel biomarker, HPV16 circE7, as a potential prognostic indicator given the unique properties of circRNA and our ability to extract it from FFPE tissue. Our study also contributes to the literature on the role of existing clinical, histologic, and immunohistochemical markers for ASCC. Larger, prospective studies are recommended to analyze the role of circE7 roles in HPV-driven cancers.

## **Materials and Methods**

### Clinical data

Patients over the age of 18 with a diagnosis of ASCC established by UT Southwestern Medical Center in Dallas, Texas between 01/01/2005 to 3/01/2019 were recruited in a retrospective, translational case series. The UT Southwestern Institutional Review Board approved this retrospective study and the need for consent was exempted. Inclusion criteria consisted of patients with a biopsy-proven diagnosis of ASCC that had follow-up at University of Texas Southwestern Medical Center. Demographic and clinical characteristics of the study cohort, including age, sex, ethnicity, comorbidities, smoking history, initial presenting symptoms, were collected through the electronic medical record. Cancer stage was based on the American Joint Committee on Cancer (AJCC) TNM classification (8th edition) [38]. Treatments were given at UT Southwestern and varied between patients. Chemotherapy consisted of 5-Fluorouracil with either Mitomycin C or Cisplatin. If surgery was performed, the methods included transanal excision, abdominoperineal resection, or pelvic exenteration. If radiotherapy was administered, it consisted of 5,040 centigray (cGy) over 28 fractional doses. Overall survival was defined as the beginning of treatment to death for any reason or the day of last follow-up.

### Bioinformatics

We downloaded raw sequencing data of cervical squamous cell carcinoma and endocervical adenocarcinoma (CESC, 309 samples) and head and neck squamous cell carcinoma (HNSC, 566 samples) from The Cancer Genome Atlas (TCGA). As the read lengths were 50-51bp, to avoid false mapping of human reads onto HPV genomes, the reads were aligned to human transcript sequences downloaded from Ensembl (release 92, GRCh38) (https://www.ensembl.org/) as well as high-risk HPV genomes and then the reads with the alignment scores on HPV genomes greater than on human transcripts were kept for the following steps for detecting circular RNA. HPV-positive samples were defined by the number of reads mapped on an HPV genome ≥10,000 and the mapping rate on the genome ≥0.00001. Based on these definitions, 125 CESC cases and 51 HNSC cases were HPV16+. The survival analysis was performed using R packages, survival (https://cran.r-project.org/web/packages/survival/) and survminer (https://cran.r-project.org/web/packages/survminer/). Kaplan–Meier survival curve of comparing high circE7 (≥30 read count and back-splice junction ratio ≥0.2) with non-circE7-high HPV+ cancers from TCGA. The GLUT1 (SLC2A1) and PD-L1 (CD274) gene expression levels were calculated by DESeq2 rlog (regularized log) after read mapping by STAR and read counting by HTSeq-count. The read ratio of E6*I (spliced isoform I) was calculated by the number of E6*I splicing reads divided by all reads covering 5’ splicing junction site.

### Immunohistochemical staining and scoring

FFPE were sectioned at 4 μm and mounted on adhesive slides, along with multi-tumor sandwich block sections containing normal and tumor tissues for external positive control and external negative control purposes. After drying the slides for 1 minute in an 1100W microwave oven set on high, the slides were transferred to a 65°C oven and dried for an additional 20 minutes. The slides were then deparaffinized in xylene and rehydrated in graded alcohols to distilled water. Endogenous peroxidase activity was quenched for 10 minutes at room temperature, using 0.3% H_2_O_2_ with 0.1% sodium azide added. Antigen retrieval was performed by placing the slides in 1 mM EDTA, pH 8.5 for 30 minutes in a household vegetable steamer, followed by a 10-minute cool-down time in the steamer. After rinsing the slides in phosphate buffered saline (PBS) buffer, primary antibody incubation was performed for 50 minutes in a 25°C incubation oven, using gentle orbital rotation at 40 rpm. Following another rinse in PBS, incubation with the appropriate anti-rabbit (for GLUT1 and PD-L1) horseradish peroxidase-conjugated polymer (PowerVision Poly-HRP anti-rabbit IgG, Leica Biosystems, Buffalo Grove, IL) was performed for 45 minutes at 25°C, using gentle orbital rotation at 40 rpm as before. Finally, the slides were immersed for 8 minutes in 25°C diaminobenzidine (DAB) (Invitrogen, Carlsbad, CA), enhanced with 0.5% copper sulfate in phosphate buffered saline (PBS) for 3 minutes at 25°C, counterstained in hematoxylin, dehydrated in graded alcohols, cleared in xylene, and coverslipped. In all cases, IHC was simultaneously performed on an in-house (ProPath) tumor tissue microarray to ensure the expected staining pattern for GLUT1 and PD-L1 stains. All controls performed appropriately.

HPV *in-situ* hybridization (HPV-ISH) shows the highest sensitivity (88%) and specificity (97%) for detecting HPV+ head and neck squamous cell carcinomas as a stand-alone assay [39]. The high-risk HPV *in situ* hybridization (HPV-ISH) probe (catalog # 312591) was obtained from Advanced Cell Diagnostics (https://acdbio.com) and was used in conjunction with their RNAscope 2.5 HD assay-brown detection kit (catalog # 322310) in accordance with the manufacturer’s instructions. Known external positive control and external negative control tissue were mounted on the same slides as the patient tissue to ensure appropriate performance of the stains. HPV-ISH screened for high-risk subtypes including 16, 18, 31, 33, 34, 35, 39, 45, 51, 52, 56, 58, 59, and 68.

Two board-certified pathologists (S.H. and G.H.) were blinded to clinical information and analyzed all histologic specimens. On H&E, the tumor architecture was recorded as “keratinizing,” “basaloid,” or “mixed.” Basaloid architecture is characterized by a solid and lobular growth pattern composed of basaloid cells with minimal cytoplasm, increased nuclear to cytoplasmic ratio, and the formation of tumor nests showing peripheral nuclear palisading with surrounding stromal fibroplasia. Keratinizing tumors consisted of tumor cells with abundant eosinophilic cytoplasm, keratinization, and the presence of prominent intercellular bridges. If discrepancies in the tumor architecture were noted between the pathologists, the sample was scored as “mixed.” The presence of perineural invasion, lymphovascular invasion, necrosis, the inflammatory response, and the tumor grade were all recorded. GLUT1 staining was measured by a weighted score, measured by the product of the percentage of stained tumor cells (0% = 0, 1–25% = 1, 26%–50% = 2, 51–75% = 3, 75%–100% = 4) and the intensity of signal (mild - 1, moderate- 2, intense - 3). High and low GLUT1 were differentiated by the median of the weighted scores. PD-L1 was measured as positive or negative with positive if >1% staining of the tumor (calculated by the sum of stained tumor cells and mononuclear cells over the total number of tumor cells). HPV-ISH was measured as positive or negative. The percentage of positive tumor cells was also recorded, as was the nature of the staining pattern (perinuclear, nuclear, cytoplasmic, or membranous).

Total RNA was extracted from FFPE slides using the Qiagen mRNAeasy kit. The extracted RNA was converted to cDNA with SuperScript IV First-Strand cDNA Synthesis Reaction using random hexamer priming. RNA levels were normalized against β-actin mRNA using back-splice specific primers for circularized E7 (Forward 5′-ACTAGGAATTGTGTGCCCCAT-3′; Reverse, 5′-GACACAGTGGCTTTTGACAGT-3′). Subjects were separated into high circE7 if the quantified mRNA was above the median value of all sample mRNA levels and low/absent circE7 if the levels were at or below the median. High and low/absent linear E6*I values were obtained similarly using the linear splice primers (Forward, 5′-GCGACGTGAGGTGTATTAAC-3′; Reverse, 5′-TAATGGGCTCTGTCCGGTTC-3′).

### Statistical analysis

Continuous data is presented as means with standard deviations and categorical data as counts with percentages. Demographic and histologic differences between biomarker levels and clinical values were compared using Pearson chi-squared for categorical data and ANOVA for continuous data. The Kaplan–Meier curves were utilized to construct the visual differences for overall survival and the biomarkers of GLUT1, PD-L1, and HPV-ISH. Univariable with the log-rank test was used to analyze factors which affect overall survival. A *p*-value of &0.05 was considered significant.

## SUPPLEMENTARY MATERIALS


