# Supplementary material for: Assessment of circularized E7 RNA, GLUT1, and PD-L1 in anal squamous cell carcinoma
Source: Oncotarget. 2019 Oct 15;10(57):5958–69. doi: 10.18632/oncotarget.27234 (PMC6800260; doi:10.18632/oncotarget.27234)
Supplement: Supplementary file 1 [file oncotarget-10-5958-s001.pdf]

Assessment of circularized E7 RNA, GLUT1, and PD-L1 in anal squamous cell carcinoma

SUPPLEMENTARY MATERIALS

A

Sample	HPV-ISH	circE7	linear E6*I
**1**	+	**1.00×10^{-7}**	9.65×10^{-5}
**2**	+	1.56×10^{-5}	1.26×10^{-2}
**3**	+	5.83×10^{-5}	**1.00×10^{-7}**
**4**	+	1.70×10^{-4}	8.08×10^{-4}
**5**	+	2.46×10^{-4}	1.24×10^{-3}
**6**	+	9.46×10^{-2}	1.05×10^{-3}
**7**	+	3.15×10^{-6}	**1.00×10^{-7}**
**8**	+	8.71×10^{-6}	3.81×10^{-4}
**9**	+	5.58×10^{-4}	1.64×10^{-3}
**10**	+	3.26×10^{-4}	1.41×10^{-3}
**11**	+	7.87×10^{-4}	5.54×10^{-5}
**12**	+	9.25×10^{-4}	1.31×10^{-3}
**13**	-	2.14×10^{-4}	3.62×10^{-6}
**14**	-	**1.00×10^{-7}**	**1.00×10^{-7}**
**15**	+	2.05×10^{-4}	7.29×10^{-4}
**16**	+	3.03×10^{-5}	8.89×10^{-3}
**17**	n.d.	2.09×10^{-4}	1.43×10^{-1}
**18**	n.d.	4.99×10^{-4}	1.49×10^{-1}

B

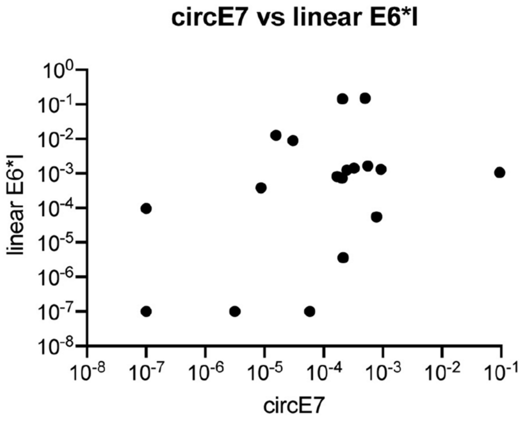

Supplementary Figure 1: HPV circE7 and linear E6*I RNA Analysis in Patients with ASCC. (**A**) Comparison of HPV-ISH and β-actin normalized circE7 and linear E6*I. Bold indicates samples with undetectable HPV16. Sample 14 was HPV-negative in all assays. (**B**) Correlation of levels of circE7 RNA and linear E6*I RNA.

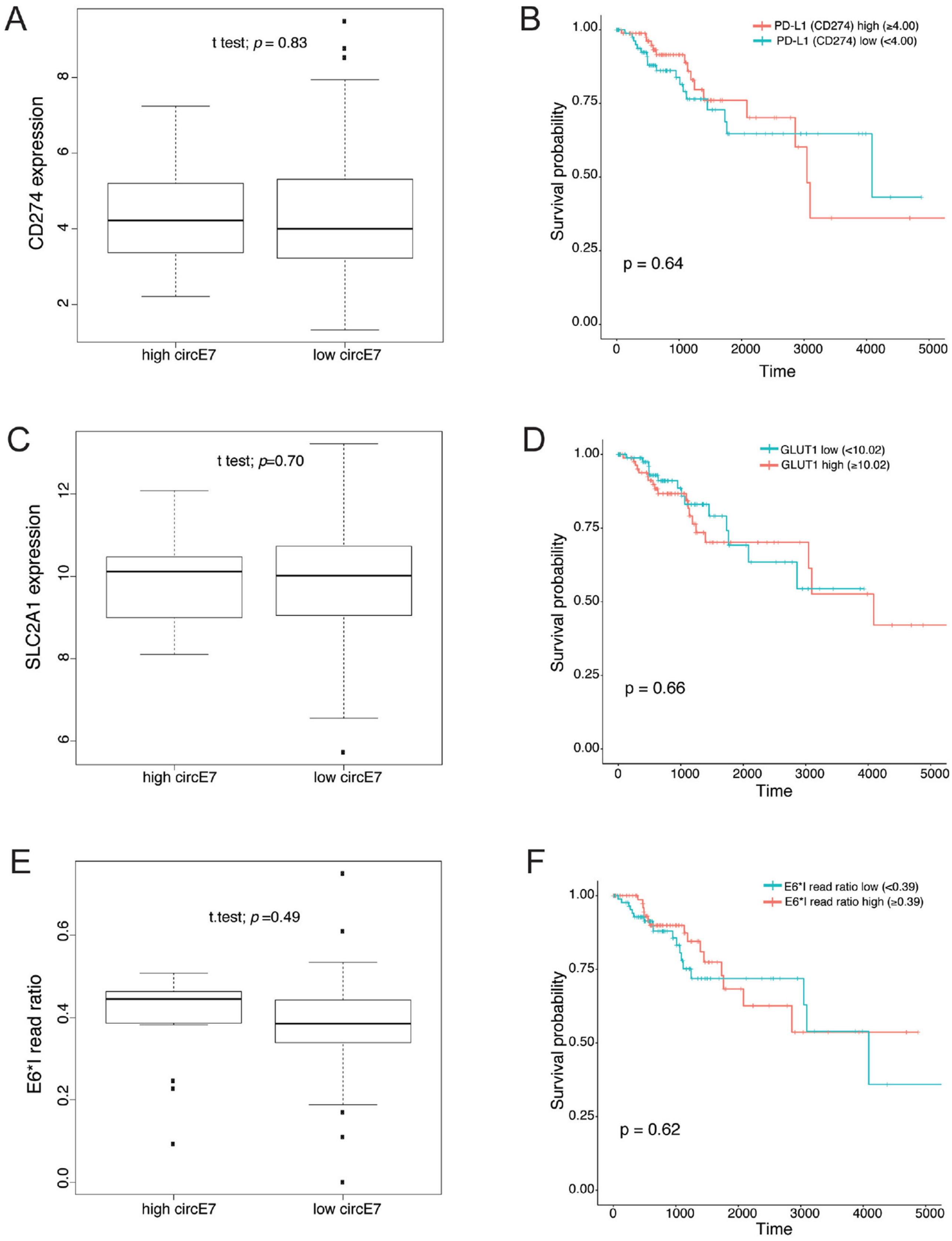

Supplementary Figure 2: Analysis of HPV+ TCGA samples. Correlation of high circE7 and low circE7 with PD-L1 expression (**A**), GLUT expression (**C**), or linear E6*I read ratio (**E**). Kaplan–Meier curves plotting overall survival based on PD-L1 expression (**B**), GLUT1 expression (**D**), or linear E6*I read ratio (**F**).

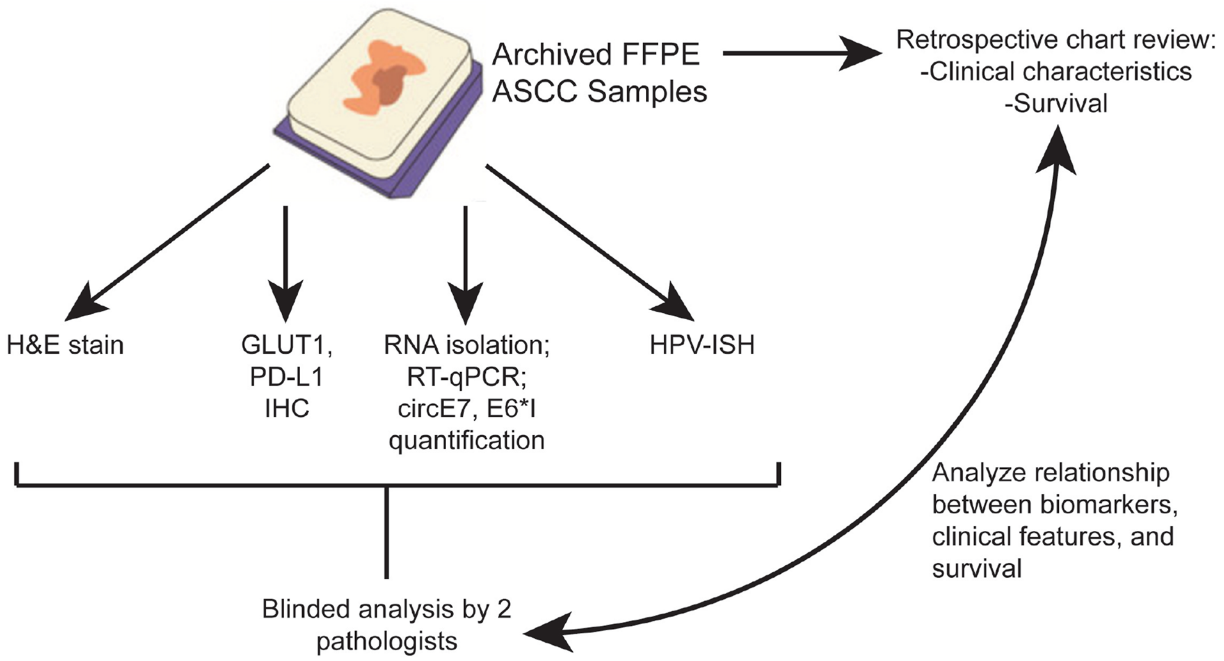

Supplementary Figure 3: Schematic of the experimental design.
